# Beyond the two compartments Petri-dish: optimising growth promotion and induced resistance in cucumber exposed to gaseous bacterial volatiles in a miniature greenhouse system

**DOI:** 10.1186/s13007-019-0395-y

**Published:** 2019-02-02

**Authors:** Geun Cheol Song, Myoungjoo Riu, Choong-Min Ryu

**Affiliations:** 1 Molecular Phytobacteriology Laboratory, KRIBB, Daejeon, 34141 South Korea; 2 Department of Applied Biology, College of Agriculture & Life Sciences, Chungnam National University, Daejeon, 34134 South Korea; 3 Biosystems and Bioengineering Program, University of Science and Technology, Daejeon, 34141 South Korea

**Keywords:** *Bacillus subtilis*, 2,3-butanediol, BVC, PGPR, ISR, Cucumber

## Abstract

**Background:**

Bacterial volatiles promote plant growth and elicit immunity responses in plants grown in two-compartment Petri dishes. Due to the limitations of bacterial volatile compound (BVC) treatments such as their high evaporation rates, it is convenient to apply BVCs in closed systems such as greenhouses. However, the concentrations of BVCs must be optimised. We therefore attempted to optimise BVC emissions from bacteria grown on solid medium and synthetic BVC treatment in order to maximise plant growth and induced resistance in a miniature greenhouse system.

**Results:**

We cultivated the model BVC emitter *Bacillus subtilis* GB03 on complex medium for continuous treatment, which we placed near 1-week-old cucumber seedlings in a miniature greenhouse. Aboveground and belowground plant growth parameters were significantly increased at 1 and 2 weeks after treatment with BVCs released by *B. subtilis* GB03. Moreover, this treatment protected cucumber seedlings against the angular leaf spot pathogen *Pseudomonas syringae* pv. *lachrymans*. In addition, cucumber shoot growth was promoted in response to the slow release of BVCs from filter paper that had absorbed 1000 and 10 µM synthetic 2,3-butanediol, a key BVC from *B. subtilis* strain GB03. However, induced resistance was only elicited when 10 plates containing 10 µM 2,3-butanediol were utilised in the miniature greenhouse. The mechanism of induced resistance appears to involve the activation of the jasmonic acid signalling pathway.

**Conclusions:**

To overcome the difficulties associated with treatment using a single application of BVC in the greenhouse, we optimised conditions for BVC application via consistent exposure in a slow-release system.

**Electronic supplementary material:**

The online version of this article (10.1186/s13007-019-0395-y) contains supplementary material, which is available to authorized users.

## Background

Since bacterial volatiles were first shown to promote plant growth, scientists have investigated the roles of bacterial volatiles as a trigger for improving plant fitness and increasing resistance to microbes [1–3]. The effectiveness of the use of bacterial volatile compounds (BVCs) under in situ conditions such as the rhizosphere has been debated because their effects were initially discovered under in vitro conditions using two-compartment Petri dishes and did not estimate in plant roots [3, 4]. To attempt to fill this gap, various studies have focused on the application of BVCs to improve plant growth and elicit induced resistance in the greenhouse and field [5–9].

For instance, when the volatile compounds 3-pentanol and 2-butanone were dissolved in water and used to treat cucumber via drench application, the plants exhibited VOC-mediated resistance against both the bacterial angular leaf spot pathogen, *Pseudomonas syringae* pv. *lachrymans*, and the aphid, *Myzus persicae*, in the open field [7]. However, most studies have demonstrated that exogenous and pharmaceutical application of BVCs to plant tissues such as roots and leaves alters plant phenotypes. The major problem in applying BVCs involves the use of synthetic chemicals rather than biologically produced BVCs from bacterial cells. It is difficult to avoid problems with biosafety when attempting to apply synthetic BVCs to plants in the field. In a recent study, BVCs produced via bacterial cultures were shown to promote plant growth and induce plant resistance [6]. BVC-mediated changes in plants represent a long-lasting effect following short-term treatment. For instance, treatment with stereoisomers of the BVC 2,3-butanediol (2,3-BDO) significantly reduced the spontaneous incidence of viruses in pepper after 90 days of treatment compared with the control. In addition, the fruit yield of plants after 100 days of 2,3-BDO treatment increased compared with the control [6]. One problem that remains to be overcome is that the diffusion rates of BVCs are too rapid to allow them to be applied directly onto crops, making it difficult to investigate their effects in the field. In most studies involving the field application of BVCs, these compounds were dissolved in water and applied to plants via drench application [6, 7]. In recent years, to avoid the rapid release of BVCs, strategies have been developed in which volatile compounds are encapsulated in biodegradable biopolymer shells to allow the volatile compounds to be released slowly over several days [10].

Once induced resistance is elicited, the response is effective for more than 20 days [11, 12]. The compelling characteristics of BVC-mediated induced resistance as a plant immune response have potential biotechnological applications for the control of diseases in crop plants under field conditions. For instance, synthetic chemical inducers such as benzothiadiazole (BTH; known as Actigard^®^ in the US and BION^®^ in Europe) [13] have been studied for their role as useful agrochemicals. BTH is highly effective at protecting plants from pathogens with minimal detrimental effects to human health and the environment. However, drench application of BTH has a critical negative effect on plant growth [14, 15]. This phenomenon, which is known as ‘allocation fitness cost’, describes the requirement for a substantial amount of metabolic resources for the manifestation of systemic acquired resistance in response to chemical elicitors, resulting in reduced plant growth [16]. BTH-treated wheat exhibits reduced growth and decreased seed production in response to chemical elicitors; the reduction in growth is more significant under nitrogen-limiting conditions [14]. However, most studies of BVC-mediated induced resistance have not uncovered a clear growth penalty in response to treatment [1, 3, 17]. A possible explanation for the elicitation of induced resistance without a growth penalty is ‘defence priming’. Early experiments aimed at eliciting induced resistance revealed that low concentrations of SA failed to trigger plant resistance but altered defence-related gene expression [18]. Defence priming is an efficient plant mechanism for acquiring immunity against multiple phytopathogens [18]. In addition, the primed state can also be prompted by rhizosphere bacteria (rhizobacteria) and entophytes [19, 20]. To apply induced resistance triggers including BVCs in the field, the need for maximum induced resistance capacity must be balanced with the need to minimise their negative effects on plant growth.

In the current study, to determine the conditions required for a minimum growth effect during the elicitation of induced resistance in a field crop, we assessed BVC-elicited induced resistance after pathogen challenge in cucumber grown in a newly established miniature greenhouse system. The objective of this study was to optimise the effects of BVCs on induced resistance and plant growth to maximise their protective effects while minimising the growth penalty in response to different amounts of BVC. To expose the plants to BVCs in the air and observe the effects of these compounds, the experiment was conducted in a newly designed miniature greenhouse system. Exposure to 20 plates of plant growth-promoting rhizobacterium (PGPR) *B. subtilis* GB03 strongly promoted plant growth but did not improve the elicitation of induced resistance. However, exposure to 10 plates of *B. subtilis* GB03 promoted plant growth while eliciting induced systemic resistance (ISR). These results were confirmed by chemical treatment with 2,3-BDO, the major BVC in strain GB03. Our results shed light on the optimal concentration of BVCs required to treat crop plants in a miniature greenhouse system rather than two-compartment Petri dishes, which have been previously used in plant–BVC interaction studies.

## Results

### Effects of different concentrations of BVCs on cucumber growth and elicitation of induced resistance

**Fig. 1:**
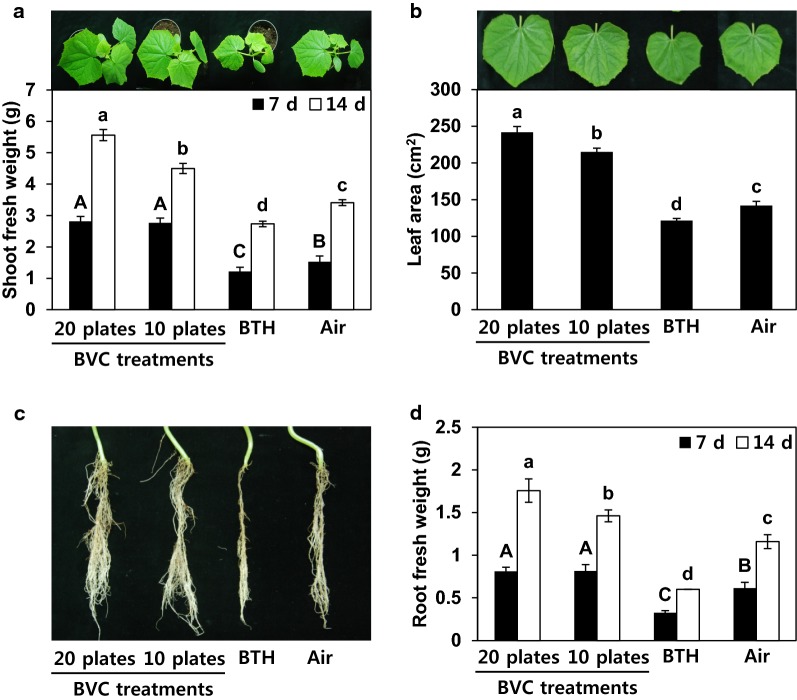
Growth promotion in cucumber seedlings in response to two difference dosages of BVCs in the miniature greenhouse system. **a** Shoot fresh weight (g) was assessed at 7 and 14 days after BVC exposure. Representative examples of cucumber seedlings after 14 days of exposure to BVCs are shown in the photograph. **b** Area of first leaf measured 14 days after BVC exposure. **c** Representative photograph of cucumber root at 14 days after BVC exposure. **d** Root weight (g) assessed at 7 and 14 days after BVC exposure. Air and 1 mM BTH were used as negative and positive controls, respectively. Different letters indicate significant differences between treatments (*P *= 0.05 according to least significant difference). Error bars indicate the standard error. Each treatment group contained four replicates (four miniature greenhouses). The experiment was repeated three times with similar results

To investigate whether BVCs can be used to enhance the growth and immunity of crops through continuous treatment, we established a miniature greenhouse system. To confer the slow release of BVCs, the bacteria were cultured on medium in stacked plates with covered lids. *B. subtilis* GB03, which was shown to release volatile compounds that promote plant growth and immunity through in vitro testing, was used as the bacterial strain in this experiment. The reactions of plants to BVCs can vary depending on the amount of volatile compounds derived from microorganisms and the size of the experimental space. We first determined that favourable conditions for promoting growth were obtained by inserting several GB03-inoculated plates into an acrylic vial (Additional file 1: Fig. S1). We investigated the interactions of BVCs and plants using *B. subtilis* GB03 grown on 10 or 20 plates. On day 7 of exposure to BVCs released from strain GB03 grown on 10 or 20 plates, the shoot weight increased more rapidly in treated plants compared with the control (Fig. 1a). On day 14 of exposure to BVCs, the shoot weight increased 1.3-fold (10 plates) and 1.6-fold (20 plates) in treated plants compared with the control (Fig. 1a). Moreover, on day 14, the size of the first leaf also increased 1.5-fold (10 plates) and 1.7-fold (20 plates) in treated plants compared with the control (Fig. 1b). Root growth increased 1.2- and 1.5-fold in response to BVC exposure from strain GB03 grown on 10 and 20 plates, respectively, compared with the control (Fig. 1c, d). The aboveground growth (shoot) in treated plants was higher than belowground growth (roots). Collectively, biologically emitted BVCs promoted cucumber growth in this miniature greenhouse system.

**Fig. 2:**
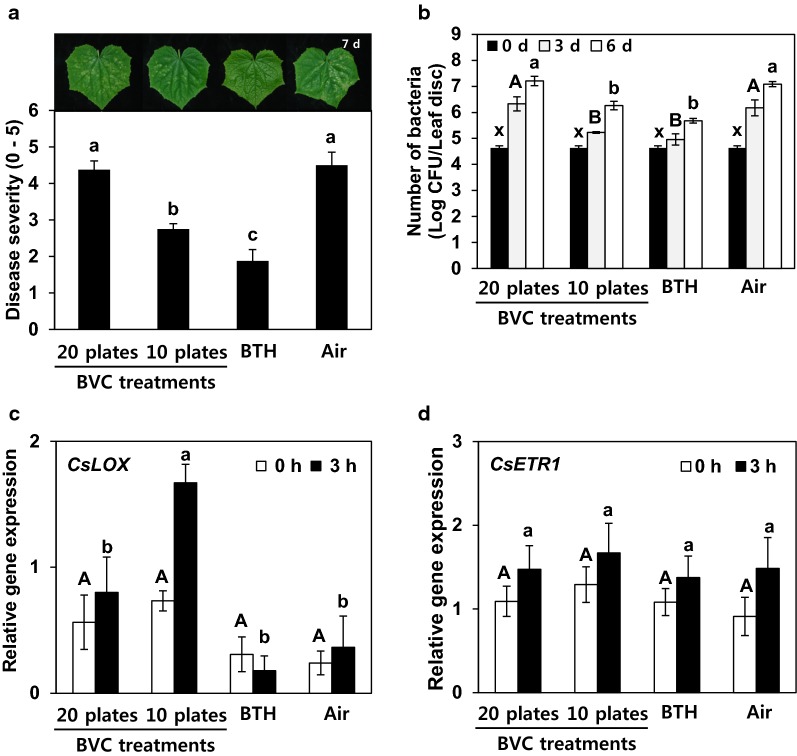
Induced resistance in cucumber seedlings in response to two difference dosages of BVCs. **a** Disease severity (0–5) in BVC-treated cucumber plants recorded at 7 days after challenge with *P. syringae* pv. *lachrymans* at OD_600_ = 1 as follows: 0, no symptoms; 1, yellowish colour; 2, chlorosis only; 3, partial necrosis and chlorosis; 4, necrosis of the inoculated area and expanded chlorosis; and 5, complete necrosis of the inoculated area. Photographs of the lesions were taken at 7 days after infection with *P. syringae* pv. *lachrymans*. **b** Pathogen population measured at 0, 3 and 6 days after challenge with *P. syringae* pv. *lachrymans* per leaf disc (diameter = 1 cm). **c**, **d** Expression levels of the cucumber resistance genes *CsLOX* and *CsETR1* assessed by qRT-PCR analysis at 0 and 3 h after challenge with *P. syringae* pv. *lachrymans*. Air and 1 mM BTH were used as negative and positive controls, respectively. Different letters indicate significant differences between treatments (*P *= 0.05 according to least significant difference). Error bars indicate the standard error. Each treatment group contained four replicates (four miniature greenhouses). The experiment was repeated three times with similar results

To determine whether the disease resistance of plants exposed to BVCs from GB03 would also be induced in the miniature greenhouse, we treated the plants with the angular leaf spot pathogen *P. syringae* pv. *lachrymans* at 14 days after exposure to the volatile compounds and measured disease severity 7 days after treatment. Interestingly, cucumber plants exposed to volatiles released from 10 plates of GB03 were resistant to the pathogen, with an average disease severity score of 2.7 (on a scale of 0–5), but plants exposed to volatiles from 20 plates of GB03 had a disease severity score of 4.2 and showed similar symptoms to the control group (Fig. 2a). When we examined the pathogen populations in the plants, plants exposed to 20 plates of GB03 appeared similar to the controls. By contrast, plants exposed to volatiles from 10 plates of GB03 exhibited reduced total pathogen populations at 3 and 6 days after inoculation compared with the control (Fig. 2b). To confirm the induction of disease resistance and defence priming by BVCs, we examined the expression of the defence-related genes *CsLOX* (for jasmonic acid signalling) and *CsETR1* (for ethylene signalling) after 0 and 3 h of pathogen challenge via qRT-PCR. Previous studies showed that volatile compounds from GB03 induce disease resistance through the jasmonic acid signalling pathway [17]. *CsLOX* transcript levels increased 2.2-fold from 0 to 3 h post-inoculation (hpi) after pre-exposure to BVCs from 10 plates of GB03, while a 1.2-fold increase was detected in control plants (Fig. 2c). On the other hand, this gene was not upregulated in cucumbers exposed to excessive levels of BVCs (from 20 plates of GB03). Unlike *CsLOX*, the expression levels of *CsETR1* did not differ among plants exposed to 10 and 20 plates of GB03 and the control (Fig. 2d).

### 2,3-BDO levels in response to different doses of *B. subtilis* GB03

**Fig. 3:**
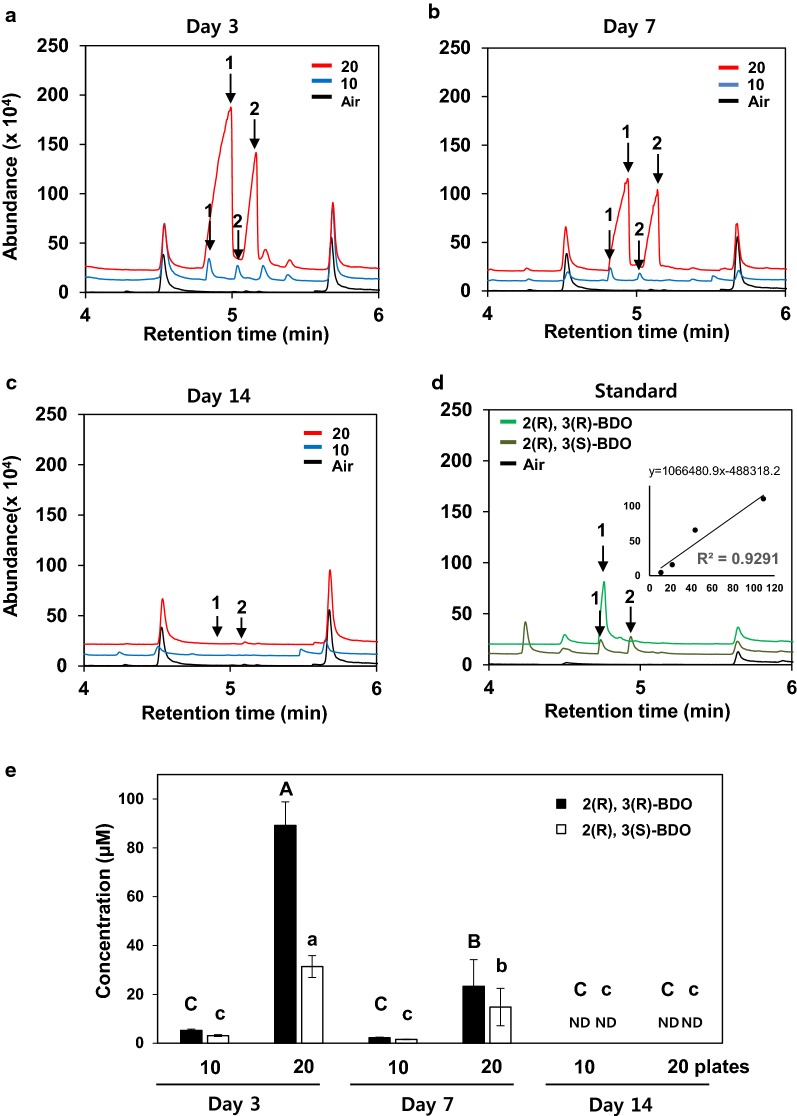
BVC profiling in the miniature greenhouse system. Chromatographic profiles of 2,3-butanediol isomer released from *B. subtilis* GB03. *B. subtilis* GB03 grown for 3 (**a**) and 7 (**b**) but not for 14 (**c**) days released 2,3-butanediol (BDO) isomers. Air = negative control treatment. Significant differences are indicated by numbers defined as follows: 2(R),3(R)-BDO (peak 1) and 2(R),3(S)-BDO (peak 2). **d** Chromatographic profiles of the synthetic chemical standard compounds 2(R),3(R)-BDO (peak 1) and 2(R),3(S)-BDO (peak 2). Air = negative control treatment. **e** Concentration of 2(R),3(R)-BDO (peak 1 in a–d) and 2(R),3(S)-BDO (peak 2 in a–d) released by *B. subtilis* GB03. (**d** inset graph). Calibration curve of 2,3-BDO. Bars represent mean ± SE. Sample size was n = 4 plants per treatment. The experiment was repeated three times with similar results

A typical volatile from GB03 that promotes plant growth is 2,3-BDO. We therefore investigated whether 2,3-BDO accumulates in the miniature greenhouse to promote cucumber growth. We collected volatile samples at three time points (3, 7 and 14 days) (Figs. 2, 3c). The highest amount of 2,3-BDO was released from GB03 on day 3 and tended to decrease over time (Fig. 3a, b). We detected 2.3-BDO from GB03 only in Meso and RR form, whereas no SS form was produced (Fig. 3c). GB03 produced 19-fold more RR-form 2.3-BDO and 12-fold more Meso-form 2.3-BDO when grown on 20 plates than when grown on 10 plates at the 3 day time point (Fig. 3c).

### Effects of different concentrations of 2,3-BDO on plant growth and elicitation of induced resistance

**Fig. 4:**
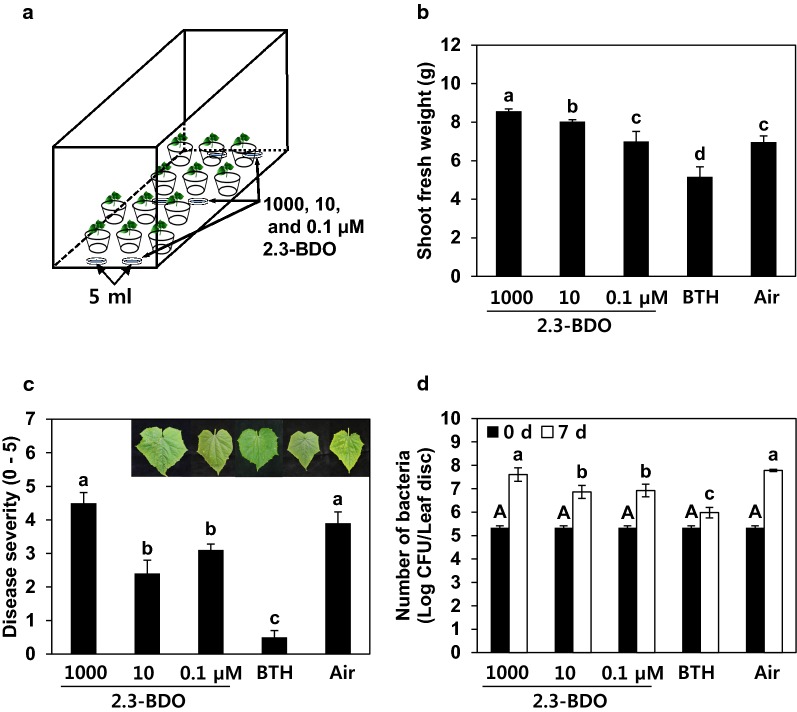
Pharmaceutical application of biologically produced BVC 2,3-butanediol to improve cucumber growth and elicit induced resistance. **a** Schematic diagram showing the experimental design to observe plant responses to different concentrations of 2,3-butanediol (2,3-BDO). The 2,3-BDO solution (1000, 10, and 0.1 μM; 5 mL total) was dispensed into plates (90 mm diameter) containing filter paper (5 × 5 cm). A total of six plates containing one piece of filter paper per plate were prepared for each concentration and placed in the miniature greenhouse together with the plants. 2,3-BDO-containing plates were placed on three different position and placed in the miniature greenhouse. **b** Shoot fresh weight (g) assessed at 14 days after 2,3-butanediol exposure. **c** Disease symptom severity (0–5) in BVC-treated cucumber plants recorded at 7 days after infection with *P. syringae* pv. *lachrymans* (10^6^ CFU/mL). Photographs of the lesions were taken at 7 days after infection with *P. syringae* pv. *lachrymans*. **d** Pathogen population measured at 0 and 7 days after challenge with *P. syringae* pv. *lachrymans*. Air and 1 mM BTH were used as negative and positive controls, respectively. Different letters indicate significant differences between treatments (*P* = 0.05 according to least significant difference). Error bars indicate the standard error (n = 6). The experiments were repeated three times with six replications

We investigated whether crop growth and immunity could be improved via the application of BVCs in an area with controlled air inflow. Specifically, we exposed cucumber plants in the miniature greenhouse system to various concentrations of 2,3-BDO and examined its effects on plant growth and immunity. As in the previous experiment, 12 plants were treated with 2,3-BDO at three concentrations (0.1, 10 or 1000 μM) (Fig. 4a), which had statistically different effects on shoot growth (Fig. 4b). Plant growth (measured as shoot weight) increased with increasing concentration of 2,3-BDO. However, plants treated with 1000 μM 2,3-BDO also lost their ability to resist disease (Fig. 4c). When treated with the angular leaf spot pathogen *P. syringae* pv. *lachrymans*, the population size of the pathogen was similar in cucumbers exposed to 1000 μM 2,3-BDO and the control (Fig. 4d). By contrast, in cucumber exposed to 10 μM 2,3-BDO, the pathogen population was reduced compared with the control; direct treatment with 2,3-BDO did not have an antagonistic effect on the pathogen (data not shown).

## Discussion

Since the initial discovery of bacterial volatile-elicited plant growth promotion and induced resistance in vitro, many studies have focused on broadening this concept and exploring its potential agricultural applications in the field [1], [5–9]. To achieve this objective, many investigators have examined the effects of spray and drench implication of liquid forms of BVCs on plants [5–9]. BVCs are not directly applied to crops due to their high volatility in open spaces, making it difficult to confirm the reproducibility of the effects of treatment with the correct concentration of BVCs and to verify the effectiveness of treatment on plants. In recent years, the cultivation of crops in greenhouses has become increasingly popular, providing a great opportunity to apply BVCs to crops while controlling the airflow around plants.

In the current study, we demonstrated that directly treating cucumber plants with BVCs in a gaseous state rather than liquid application had a strong growth-promoting effect and provided strong protection against a bacterial pathogen under miniature greenhouse conditions (Figs. 1, 2). The successful results obtained using plants grown in vitro in a small-scale Petri dish containing two compartments (I-plate) prompted us to expand BVC usage to a larger closed system, i.e., the miniature greenhouse. In addition, we performed translational research by investigating a crop (cucumber) rather than a model plant. When developing ways to apply BVCs in the field, a major concern is to prevent the BVCs from rapidly volatilising. One proposed method is to encapsulate volatile compounds in biodegradable biopolymer shells to allow the volatile compounds to be released slowly over several days [10]. However, this method is still being developed, and many experiments are needed before it can be applied to the field. Here, we investigated the use of volatiles to protect a greenhouse-grown crop plant. Ten or twenty plates of BVC-emitting *B. subtilis* GB03 were stacked to physically block the rapid volatilisation of the BVCs. Continuous exposure of plants to BVCs from 10 bacterial culture plates provided the maximum levels of both plant growth promotion and induced resistance. Our data suggest that new technology can be developed to expose aboveground plant parts to BVCs to improve plant health.

We attempted to optimise plant growth and maximise induced resistance because increased plant growth is not always linked with enhanced plant immunity [15, 21]. In the current study, plant growth (as indicated by increased shoot weight, root weight and leaf area) was significantly enhanced in plants at 7 and 14 days after exposure to BVCs from 20 plates of *B. subtilis* GB03 on culture medium (Fig. 1). However, no significant disease protection against *P. syringae* pv. *lachrymans* and no clear defence priming of cucumber defence-related genes *CsLOX* and *CsETR1* was detected under these conditions (Fig. 2). Treatment with 50% fewer BVCs (10 plate treatment) elicited both growth promotion and induced resistance (Figs. 2, 3). Methods that make use of the dual effects of BVCs rather than a single effect have greater potential for practical application.

The continuous application of BVCs in a closed greenhouse has not previously been investigated. However, when the effects of single applications of BVCs were tested, growth promotion and plant resistance were sometimes simultaneously induced, while some treatments only had a single effect. Drench application of cucumber plants with the bacterial volatile, 2-butanone, has been successfully performed for several years under field conditions [7]. Two concentrations of 2-butanone (0.1 and 10 µM) mediated induced resistance against both the bacterial angular leaf spot pathogen, *P. syringae* pv. *lachrymans*, and the sucking insect aphid, *Myzus persicae*, in the open field. However, these treatments did not increase shoot length or fresh weight compared with the control. When pepper plants were subjected to drench application of 2,3-BDO, a significant reduction in the incidence of naturally occurring viruses (*Cucumber mosaic virus* and *Tobacco mosaic virus*) was observed compared with the control [7]. The effect of 2,3-BDO on pepper growth was not investigated in this study, but treatment with 2,3-BDO was shown to promote disease resistance and plant growth in *Arabidopsis* [6].

BTH, which is often used as a positive control in experiments examining induced resistance, consistently confers resistance against plant pathogens, but inhibits growth compared with the control [22]. This phenomenon is referred to as ‘allocation fitness cost’ or ‘trade-off’ [21]. The reduction in growth is due to the competition for plant-related compound biosynthesis/metabolism and the significant demand for energy for induced resistance [23]. However, in the current study, the induction of systemic resistance by BVC did not show any growth penalty (Fig. 1a, b). In the majority of cases, BTH increases the expression of defence-related genes in the absence of pathogens, while BVC induces the immediate expression of resistance genes only after the plant is exposed to a pathogen [15, 24]. The induction of the defence response is more rapid and stronger only at the moment the plant senses the elicitor or pathogen, a process known as ‘defence priming’ [15]. In the current study, the elicitation of plant immunity was validated by the defence priming of *CsLOX*, a marker gene for jasmonic acid signalling following pathogen challenge, in BVC-treated cucumber leaves (Fig. 2c). Our findings suggest that jasmonic acid plays a critical role in BVC-mediated induced resistance against *P. syringae* pv. *lachrymans*, while ethylene signalling might not be involved in this process (Fig. 2c, d).

In addition to testing the effects of BVC-emitting bacterial cultures in a miniature greenhouse, we performed profiling of 2,3-BDO in our system over the course of treatment (Fig. 3). 2,3-BDO has been identified as a representative BVC in many studies [1, 17, 25, 26]. Drench application of *Arabidopsis* and pepper plants with 2,3-BDO is effective at inducing resistance. When *Arabidopsis* was treated with 2,3-BDO, only 2R, 3R-BDO isomers were used [17]. Here, we analysed the isomer forms from BVCs and detected two stereoisomers: 2(R),3(R)-BDO and the Meso form of 2,3-BDO (Fig. 3). Both stereoisomers promoted plant growth and induced resistance. However, the effective concentration varied depending on the isomer. A recent study examined the optimal concentration of 2,3-BDO for use to control cucumber mosaic virus and tobacco mosaic virus in the field and whether the effect of this compound differed depending on the isomer. In field studies, treatment with 2R, 3R-BDO and 2R, 3S-BDO significantly reduced the incidence of spontaneous virus compared with 2S, 3S-BDO and control treatments [6]. In the current study, the highest 2,3-BDO levels were detected at 3 days and gradually decreased thereafter (Fig. 3c). Our system can be potentially used to apply 2,3-BDO in greenhouses. We propose that 89 and 5.2 µM are the minimum concentrations of 2,3-BDO required to promote plant growth and elicit induced systemic resistance in our system, respectively (Fig. 3e). These results indicate that the initial exposure to high levels of BVCs is critical for eliciting plant responses.

To optimise treatment with BVCs, we also tested the effects of different concentrations of 2,3-BDO (biologically synthesised in bacterial cells) in plants in the miniature greenhouse system. Although it is difficult to consistently treat plants with a specific amount of a single concentration of 2,3-BDO, we successfully identified a concentration that promoted growth in plants treated with various concentrations of this compound (100-fold difference from 0.1 to 1000). Similar to the results obtained using a higher number of plates, the highest concentration of 2,3-BDO tested (1000 μM) had the greatest growth-promoting effect on cucumber shoot weight (Fig. 4b) but did not increase plant immunity (Fig. 4c). However, treatment with a moderate concentration of 2,3-BDO (10 μM) had the optimal plant protective effect against *P. syringae* pv. *lachrymans* while still promoting plant growth. Indeed, in previous studies, all concentrations of 2,3-BDO below 0.22 mM were shown to induce resistance in plants. However, this compound increased plant growth only at intermediate concentrations. Additional studies are needed to explain why the effects of this compound, particularly on mRNA expression of *CaLOX*, vary according to concentration at the molecular level.

Our findings suggest that biochemical treatment can improve plant immunity without a growth penalty, instead promoting plant growth. This concept could have great applications to agriculture. However, before BVCs can be routinely applied in the field, many other issues should be considered, such as human toxicity and application strategies. BVCs have great potential for use in plant stress management. However, due to the nature of these compounds, they are prone to evaporation or reactions with other compounds, highlighting the need to develop effective formulations for commercial use. New techniques for converting volatile substances into natural insecticides were recently described [2, 27]. Briefly, volatiles are encapsulated in a biodegradable biopolymer shell to enable their slow release over several days. These formulations can be applied to farms or greenhouses using traditional sprayers or seeders.

## Conclusions

Plants can be exposed to volatiles in the air in a miniature greenhouse to promote growth and improve disease resistance. These effects can occur separately or simultaneously, depending on BVC concentration. Changes in the global environment are prompting growers to cultivate crops in the greenhouse rather than the open field to ensure safe crop production. The successful treatment of plants with volatiles in the air should have a marked effect on greenhouse cultivation. The results of this study lay the foundation for the practical agricultural use of volatile compounds.

## Methods

### Plant growth and treatment with Bacillus subtilis GB03 VOCs

Cucumber seeds (*Cucumis sativus* L. cv. backdadagi, Nongwoobio, Co. Ltd., Suwon, South Korea) were surface sterilised with 3% sodium hypochlorite, washed four times with sterilised distilled water and sown on autoclaved soil-less potting medium (Punong, Co. Ltd., Gyeongju, South Korea) containing zeolite, perlite, colour dust and lime (pH ranging from 4.5 to 7.5). The cucumber plants were cultivated in a growth chamber at 28 °C under a 16 h (h)/8 h light/dark photocycle. The miniature greenhouse was 55 cm wide, 95 cm long and 65 cm high, and was designed to block external air circulation. Each pot contained a single cucumber plant, with 12 pots per miniature greenhouse. *B. subtilis* GB03 was grown on solid tryptic soy agar (TSA) containing 100 µg/mL rifampicin at 30 °C for 1 day and adjusted to the proper concentration (OD_600_ = 1) in 10 mM MgCl_2_. As a positive control, 30 mL of 1 mM BTH per pot was drench-applied to cucumber roots. Measurements of plant growth and disease parameters at each time point are briefly described below.

1. *Week 0*, 12 seedlings were transferred to a custom-made miniature greenhouse at 1 week after sowing for exposure to BVCs released by *B. subtilis* GB03. *B. subtilis* GB03 was grown on solid TSA containing 100 µg/mL rifampicin at 30 °C for 1 day. Ten or twenty plates were placed in the miniature greenhouse.
2. *Week 1*, the shoot and root fresh weights and leaf area of cucumber plants in each treatment group were measured. To measure the total leaf area, the first true leaf was collected and image analysis was performed using Assess V2.0: Image Analysis Software (APS Press, c2002St. Paul, MN, USA).
3. *Week 2*, the shoot and root fresh weights and leaf area of cucumber plants in each treatment group were measured. To avoid direct effects of BVC exposure on plant pathogens, the plates were removed prior to pathogen challenge. For pathogen challenge and disease assessment, *P. syringae* pv. *lachrymans* was cultured overnight at 28 °C in King’s B medium supplemented with 100 μg/mL rifampicin. Seedling leaves were sprayed with a culture of *P. syringae* pv. *lachrymans*, which was suspended in 10 mM MgCl_2_ such that OD_600_ = 1 until run-off on cucumber seedling leaves at 14 days after BVC exposure [28]. Plants were returned to the growth chamber immediately after inoculation.
4. *Week 3*, BVC-mediated induced resistance to *P. syringae* pv. *lachrymans* was evaluated at 7 days after inoculation. Disease severity was scored as follows: 0, no symptoms; 1, less than 20% of the whole leaf was diseased; 2, 21–40% of the whole leaf was diseased; 3, 41–60% of the whole leaf was diseased; 4, 61–80% of the whole leaf was diseased; and 5, more than 81% of the whole leaf was diseased [4]. The number of bacteria per leaf disc (diameter = 1 cm) was counted according to previously described methods [28]. Leaves were harvested at 0 and 3 h after inoculation and immediately frozen in liquid nitrogen for total RNA extraction. Intact cucumber leaves were used for the non-stress treatments. The experiment had 12 replicates, with one plant per replicate. The experiment was repeated three times with similar results.

### Pharmaceutical application of 2,3-BDO in the miniature greenhouse system

Filter paper discs in Petri dishes (90 mm diameter) were soaked with 5 mL of 1000, 10 or 0.1 µM 2,3-BDO (Sigma-Aldrich; 2(R),3(S)-butanediol, CAS no. 513-85-9; 2(R),3(R)-butanediol, CAS no. 24347-58-8, Daejeon, South Korea). Six plates containing filter paper were prepared for each concentration of 2,3-BDO and placed in a miniature greenhouse together with the plants. Considering the differences in diffusion rates of 2,3-BDO, the 2,3-BDO-containing plates were divided into three parts and placed in a miniature greenhouse. Growth and disease resistance parameters were measured in the plants after 2 weeks of exposure. Each treatment group included four replicates (four miniature greenhouses). The experiment was repeated three times with similar results.

### Measurement of 2,3-BDO levels

*Bacillus subtilis* GB03 was cultured aerobically at 28 °C in a miniature greenhouse. After 3 days of growth, a solid-phase microextraction fibre (SPME; divinylbenzene/carboxen/polydimethylsiloxane, 50/30 µm; Supelco/Sigma-Aldrich, St. Louis, MO, USA) was inserted into the miniature greenhouse and exposed for 1 h. All fibres were conditioned in the GC injection port before use following the manufacturer’s guidelines. All fibres were handled with a manual holder. Separation was achieved using the following temperature programme: initial temperature of 50 °C with a 2 min hold, ramped up to 220 °C at 10 °C/min and held for 2 min. The split–splitless injection port was held at 280 °C for the desorption of volatiles in split mode at a split ratio of 1:10. Helium was used as the carrier gas at a constant flow rate of 1.0 mL/min. An HP-5MS 30 m × 0.25 mm × 0.25 µm (Hewlett Packard) non-polar column was used. The separation of VOCs was achieved using the following temperature program: an initial temperature of 40 °C with a 3 min hold, ramped up to 220 °C at 10 °C/min and held for 2 min. The split–splitless injection port was held at 250 °C in splitless mode. The MS parameters were as follows: full-scan mode with a scan range of 40–500 amu at a rate of 0.817 scan/s. The ion source temperature was 250 °C with an ionising energy of 70 eV and a mass transfer line temperature of 300 °C. VOC identification was achieved using the National Institute of Standards and Technology (NIST) reference library and a comparison of the retention times (tR) and mass spectra of authentic standards. In addition, an in-house dedicated mass spectral library was built using the mass spectra of authentic compounds to confirm the identities of the detected VOCs [29, 30]. A calibration curve generated using three concentrations of an external standard was used to quantify the levels of 2,3-BDO in miniature greenhouses (Fig. 3d inset). The correlation coefficient was 0.929 (Fig. 3d). Levels of 2,3-BDO were calculated using the equation obtained from the calibration curve [30, 31].

### Extraction of plant RNA, cDNA synthesis and quantitative reverse-transcription PCR

Following inoculation with pathogen, the plants were returned to the growth chamber, and leaf tissue was harvested 0 and 3 h after inoculation with *P. syringae* pv. *lachrymans* and used for total RNA isolation. Total RNA was isolated using an RNeasy^®^ Plus Mini Kit according to the manufacturer’s protocol (Qiagen, USA). First-strand cDNA was synthesised using 2 µg of RNA, oligo dT primer, dNTP and Moloney murine leukaemia virus reverse transcriptase (M-MLV RT; Enzynomics, Daejeon, South Korea). Quantitative reverse-transcription PCR (qRT-PCR) was carried out using a Chromo4 real-time PCR system (Bio-Rad, CA, USA). The reaction mixture contained 2 × Brilliant SYBR Green qRT-PCR Supermix (Bio-Rad), cDNA and 0.5 µM of each gene-specific primer. The expression levels of candidate priming genes were analysed using the following primers: *Cucumis sativus Lipolygenase* (*CsLOX*), forward 5′-AAGGTTTGCCTGTCCCAAGA-3′ and reverse 5′-TGAGTACTGGATTAACTCCAGCCAA-3′; *C. sativus Ethylene Receptor* (*CsETR*), forward 5′-GCCATTGTTGCAAAAGCAGA-3′ and reverse 5′-GCCAAAGACCACTGCCACA-3′; *C. sativus Actin* (*CsActin*), forward 5′-CCGTTCTGTCCCTCTACGCTAGTG-3′ and reverse 5′-GGAACTGCTCTTTGCAGTCTCGAG-3′ [28]. Relative RNA levels were calibrated and normalised relative to the level of *CsActin* mRNA. The sequences were amplified using the following thermocycler parameters: 10 min at 95 °C, followed by 44 cycles of 30 s at 95 °C, 30 s at 60 °C and 42 s at 72 °C.

### Statistical analysis

Analysis of variance of the experimental datasets was performed using JMP software version 5.0 (SAS Institute Inc., Cary, NC, USA; www.Sas.com). Significant effects of treatment were determined based on the magnitude of the *F*-value (*P* = 0.05). When a significant *F*-test was obtained, separation of means was accomplished by Fisher’s protected LSD at *P* = 0.05.

## Additional file

**Additional file 1: Figure S1.** Schematic representation of the experimental design using the miniature greenhouse system. (a) The miniature greenhouse, which is 55 × 95 × 65 cm in size, blocks external air circulation. Twelve pots were placed in the miniature greenhouse. (b) Representative photographs taken at 14 days after BVC exposure. Each treatment group contained four replicates (four miniature greenhouses). (c) Measurement of growth and induced resistance at 0, 1, 2 and 3 weeks after BVC exposure. Week 0, 12 seedlings were transferred to a custom-made miniature greenhouse at 1 week after sowing for exposure to BVCs released by *B. subtilis* GB03. Week 1, the shoot and root fresh weights and leaf area of cucumber plants in each treatment group were measured. Week 2, the shoot and root fresh weights and leaf area of cucumber plants in each treatment group were measured. To avoid direct effects of BVC exposure on plant pathogens, the plates were removed. Thereafter, seedling leaves were sprayed with a culture of *P. syringae* pv. *lachrymans*, which was suspended in 10 mM MgCl2 such that OD_600_ = 1. Plants were returned to the growth chamber immediately after inoculation. Week 3, BVC-mediated induced resistance to *P. syringae* pv. *lachrymans* was evaluated at 7 days after inoculation. Disease severity was scored as follows: 0, no symptoms; 1, less than 20% of the whole leaf was diseased; 2, 21–40% of the whole leaf was diseased; 3, 41–60% of the whole leaf was diseased; 4, 61–80% of the whole leaf was diseased; and 5, more than 81% of the whole leaf was diseased. Leaves were harvested at 0 and 3 h after inoculation and immediately frozen in liquid nitrogen for total RNA extraction. Intact cucumber leaves were used for the non-stress treatments. The experiment had 12 replicates, with one plant per replicate. The experiment was repeated three times with similar results.
