# Supplementary material for: Beyond the two compartments Petri-dish: optimising growth promotion and induced resistance in cucumber exposed to gaseous bacterial volatiles in a miniature greenhouse system
Source: Plant Methods. 2019 Feb 2;15:9. doi: 10.1186/s13007-019-0395-y (PMC6359797; doi:10.1186/s13007-019-0395-y)
Supplement: Supplementary file 1 — Additional file 1: Figure S1. Schematic representation of the experimental design using the miniature greenhouse system. (a) The miniature greenhouse, which is 55 × 95 × 65 cm in size, blocks external air circulation. Twelve pots were placed in the miniature greenhouse. (b) Representative photographs taken at 14 days after BVC exposure. Each treatment group contained four replicates (four miniature greenhouses). (c) Measurement of growth and induced resistance at 0, 1, 2 and 3 weeks after BVC exposure. Week 0, 12 seedlings were transferred to a custom-made miniature greenhouse at 1 week after sowing for exposure to BVCs released by B. subtilis GB03. Week 1, the shoot and root fresh weights and leaf area of cucumber plants in each treatment group were measured. Week 2, the shoot and root fresh weights and leaf area of cucumber plants in each treatment group were measured. To avoid direct effects of BVC exposure on plant pathogens, the plates were removed. Thereafter, seedling leaves were sprayed with a culture of P. syringae pv. lachrymans, which was suspended in 10 mM MgCl2 such that OD600 = 1. Plants were returned to the growth chamber immediately after inoculation. Week 3, BVC-mediated induced resistance to P. syringae pv. lachrymans was evaluated at 7 days after inoculation. Disease severity was scored as follows: 0, no symptoms; 1, less than 20% of the whole leaf was diseased; 2, 21–40% of the whole leaf was diseased; 3, 41–60% of the whole leaf was diseased; 4, 61–80% of the whole leaf was diseased; and 5, more than 81% of the whole leaf was diseased. Leaves were harvested at 0 and 3 h after inoculation and immediately frozen in liquid nitrogen for total RNA extraction. Intact cucumber leaves were used for the non-stress treatments. The experiment had 12 replicates, with one plant per replicate. The experiment was repeated three times with similar results. [file 13007_2019_395_MOESM1_ESM.pptx]

## Slide 1
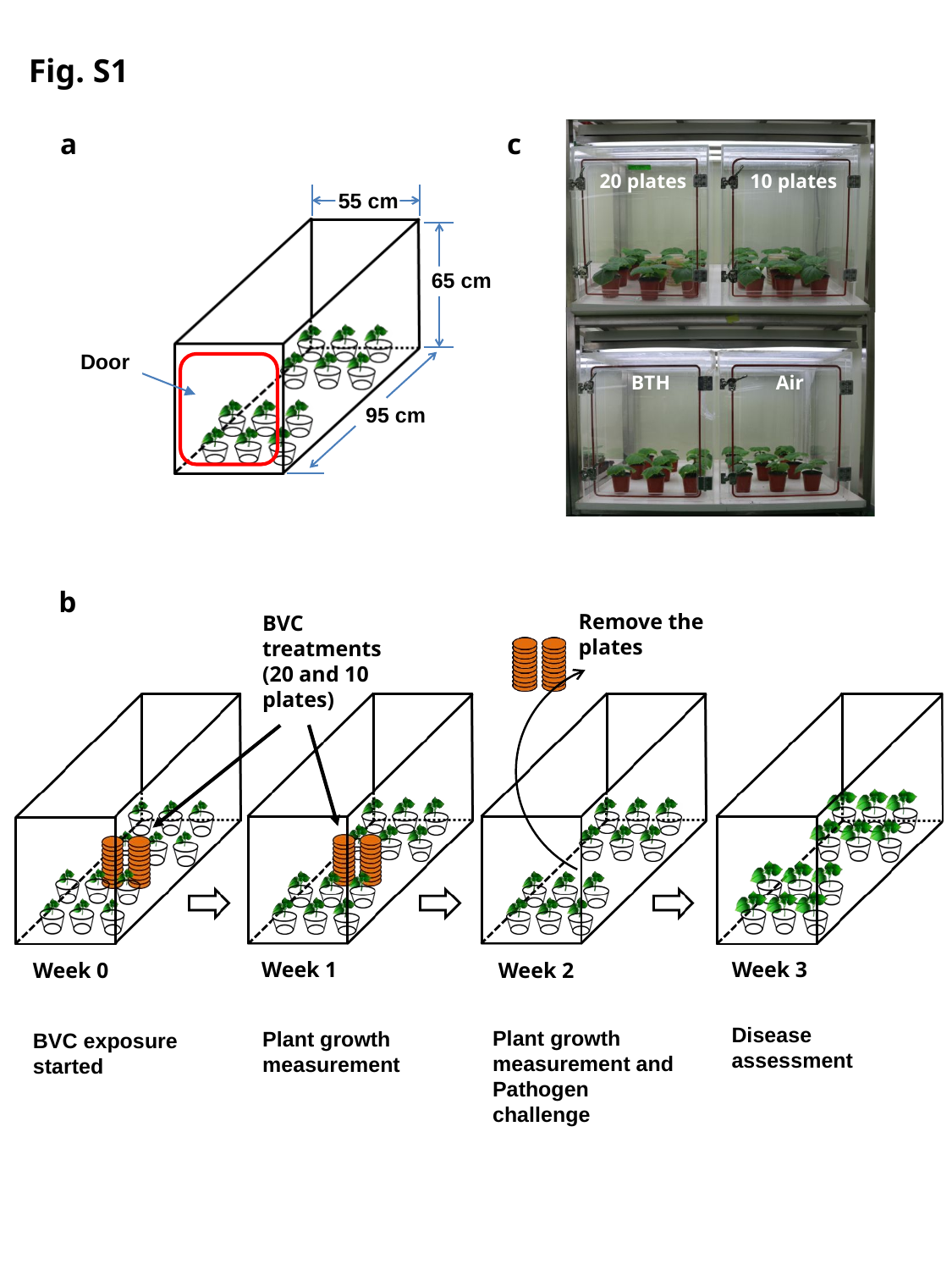

Fig. S1
a
c
20 plates
10 plates
BTH
Air
55 cm
65 cm
Door
95 cm
b
Remove the plates
BVC treatments
(20 and 10 plates)
Week 3
Week 1
Week 2
Week 0
Disease assessment
Plant growth measurement and
Pathogen challenge
Plant growth measurement
BVC exposure started
